# Clinical diagnosis and endoscopic analysis of 10 cases of intestinal tuberculosis

**DOI:** 10.1097/MD.0000000000021175

**Published:** 2020-07-10

**Authors:** Shuangshuang Lu, Jinjin Fu, Yongxin Guo, Jin Huang

**Affiliations:** a School of Medical, Dalian Medical University, Dalian; b Changzhou Second People's Hospital of Nanjing Medical University, Changzhou, China.

**Keywords:** intestinal tuberculosis, clinical analysis, endoscopy, pathology

## Abstract

To analyze the clinical characteristics of intestinal tuberculosis (ITB), pay attention to the diagnostic value of endoscopy and mucosal biopsy, improve the recognition of atypical manifestations of ITB under endoscopy, and reduce misdiagnosis and missed diagnosis.

The clinical data of 10 patients who were hospitalized in Changzhou second people's Hospital and finally diagnosed as ITB from January 1, 2015 to present were analyzed retrospectively. The basic information, medical history, clinical manifestations and computed tomography (CT), endoscopy of the patients was analyzed retrospectively. The results of pathological examination were analyzed and sorted out.

Among the 10 patients, the ratio of male to female was 7:3, 10 (100%) had abdominal pain, 3 (30%) had diarrhea and 2 (20%) had bloody stool. The positive rate of tuberculosis T cell test was 75% (6/8), the diagnostic rate of chest high resolution CT was 60%, and the abnormal rate of abdominal high-resolution CT was 66.7% (6/9). Colonoscopy showed that the lesions mainly involved ileocecum (70%) and ascending colon (60%). Most of the lesions were intestinal stenosis (60%) and circular ulcer (50%). In a few cases, cold abscess (20%) and scar diverticulum (10%). Most of the pathological manifestations were granuloma formation and multinucleated giant cells (60%). The detection rate of caseous granuloma was 20%.

The general condition and clinical manifestations of patients with ITB are not specific. Endoscopy and mucosal biopsy are of great significance for its diagnosis. The clinical manifestations and endoscopy of some patients showed atypical signs. Therefore, the combination of multi-disciplinary team models and the enhancement of clinician's recognition of the characteristics of endoscopic examination of ITB can improve us the diagnosis level of ITB.

## 1 Introduction

Intestinal tuberculosis (ITB) invading the intestinal wall is a chronic specific infection caused by Mycobacterium tuberculosis, which is mostly secondary to extrapulmonary tuberculosis, especially pulmonary tuberculosis.^[1]^ Gastrointestinal tuberculosis accounts for about 0.15% of gastrointestinal infection.^[2]^ Statistics show that 58% of tuberculosis (TB) patients in the UK have extrapulmonary diseases and 5.9% have ITB.^[3]^ In recent years, due to the worldwide incidence of pulmonary tuberculosis is increasing, the incidence of ITB is also on the rise. Due to the lack of specificity in clinical manifestations and laboratory examination of ITB, it is easy to cause misdiagnosis.^[4]^ Once misdiagnosis affects the prognosis of patients, the economic burden will increase; More seriously, missed diagnosis will lead to the miss of the early treatment of the disease, and it will be more difficult to control the development of the disease.^[5,6]^ Therefore, it is urgent to improve the clinical diagnosis and treatment of ITB. In this paper, the clinical data of 10 patients with ITB treated in our hospital from January 1, 2015 to January 1, 2020 were analyzed retrospectively, and their clinical, radiologic, endoscopic and histological features were analyzed in order to provide experience and reference for improving the diagnosing of ITB.

## 2 Data and Methods

### 2.1 General Information

The clinical data of 10 patients who were hospitalized in Changzhou second people's Hospital and finally diagnosed as ITB from 2015 to present were collected and analyzed retrospectively. General data include: date of hospitalization, sex, age, occupation, current address, place of origin, date of first visit, date of diagnosis, etc. Clinical data include: medical history, clinical manifestations, image, endoscopic and pathological characteristics, therapeutic drugs, therapeutic effects, such as surgery, but also including intraoperative and postoperative pathological examination.

### 2.2 Research Methods

Taking the time of admission as the starting point and the end point of the study on September 1, 2019, the patients were followed up by telephone and outpatient visit for not less than 6 months. The clinical manifestations, image, endoscopic and pathological characteristics and treatment outcome of 10 cases who met the inclusion criteria^[7]^ were analyzed retrospectively, and then summarized.

All patients agreed for conservative management. Written informed consent for endoscopy and participation in the study were obtained from patients or near relatives. The study protocol was approved by the ethics committees of Changzhou Second People's Hospital of Nanjing Medical University.

### 2.3 Statistical Method

The data were statistically analyzed by excel2019 software.

## 3 Result

### 3.1 General Data Analysis

The general data of 10 patients were analyzed. Results are shown as Table 1.

**Table 1:**
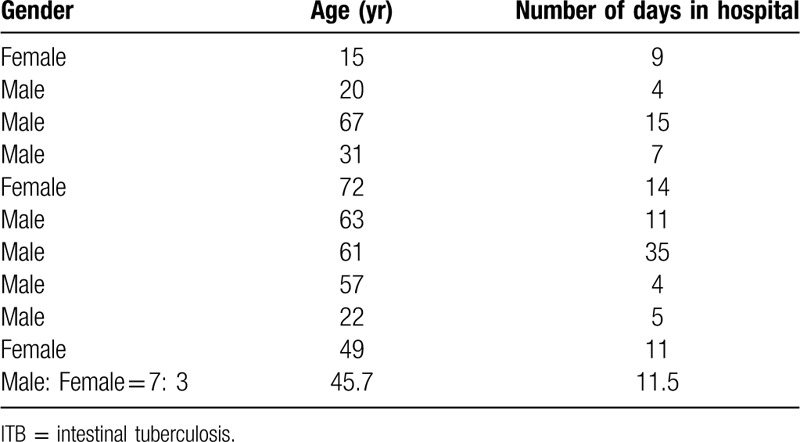
General data of 10 patients with ITB.

### 3.2 Clinical Manifestations

The main clinical manifestations of 10 patients were abdominal pain, diarrhea, wasting, followed by abdominal distention, cough, expectoration, abdominal mass, etc. Among them, abdominal pain is mainly lower abdominal pain or periumbilical dull pain, which is paroxysmal; diarrhea is often irregular loose stool, 3 to 5 times/d, often accompanied by mucus and undigested food, without pus blood, occasionally with blood in the stool. They are shown as Table 2.

**Table 2:**
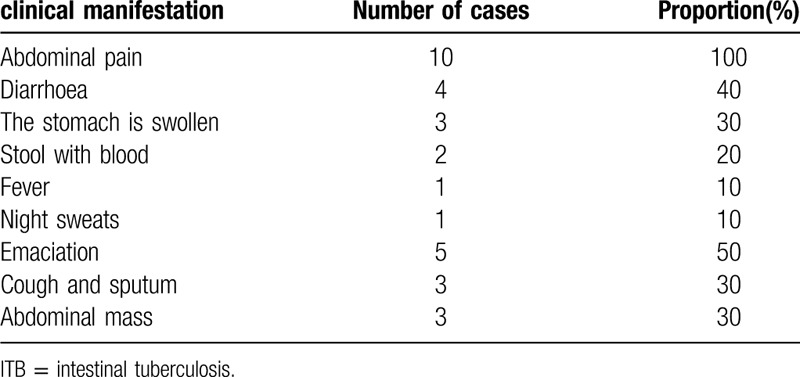
Clinical manifestations of 10 patients with ITB.

### 3.3 Laboratory Examination and Imaging Examination

Of the 10 patients, 8 patients underwent T-spot test, 6 patients were positive, and 1 of the patients who did not do the test was non-first-time pulmonary tuberculosis. All of them had a high-resolution chest computed tomography (CT), and 2 patients had a chest X-ray at the same time. At the same time, 9 patients underwent abdominal high-resolution CT examination, of which 6 patients had partial intestinal wall thickening, 4 patients had multiple lymph nodes and pelvic effusion in abdominal cavity and retro peritoneum, and 3 patients had no obvious abnormality in the structure and morphology of abdominal intestines. Results are shown as Table 3.

**Table 3:**
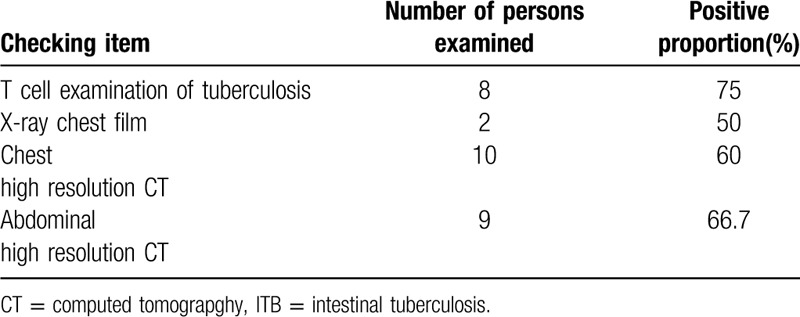
Laboratory and imaging examinations of 10 patients with ITB.

Colonoscopy and Pathological Examination

### 3.4 Endoscopic Findings of ITB

**Figure 1:**
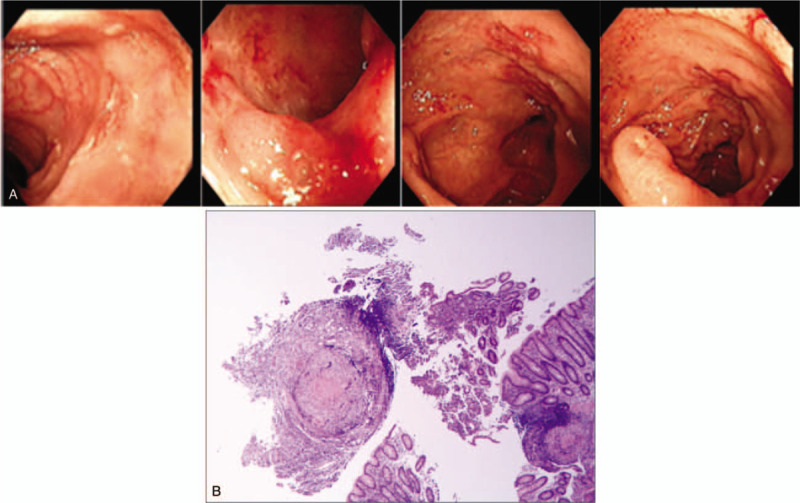
A. Multiple ulcers of different sizes can be seen in the mucosa of terminal ileum, cecum and ileocecal flap. B. Ascending colon ulcers, local granulomatous inflammation, multi nuclear giant cell reaction and a small amount of coagulative necrosis.

**Figure 2:**
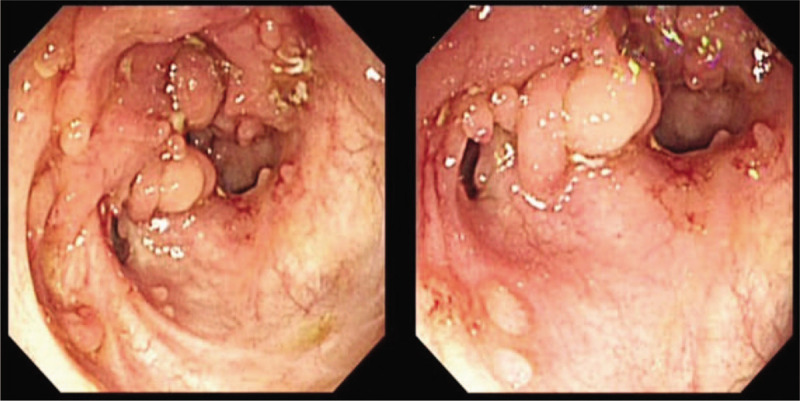
Ulcerative lesions were seen in the ascending colon, with irregular hyperplasia and narrow intestinal cavity.

**Figure 3:**
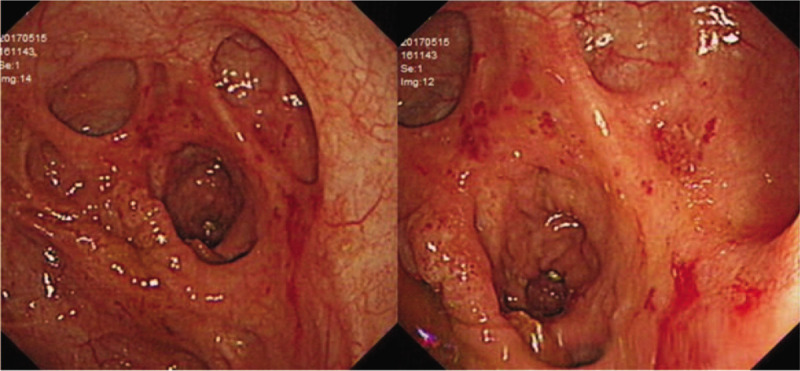
Scar diverticulum of ileocecum.

Colonoscopy was performed in all the 10 patients who were included in the study. Endoscopic findings of ITB show that the lesions mainly involve the ileocecum and ascending colon, with the characteristics of continuous lesions, varying in size, irregular edges showing rat bite sign, ulcers are mostly annular ulcers (Fig. 1A), and most patients have intestinal stenosis (Fig. 2). In a few cases, scar diverticulum (Fig. 3) and cold abscess can be seen, which will certainly attract our attention. At the same time of endoscopy as shown in Figure 1A, two pieces of millet sized tissue and 1 piece of needle tip sized tissue in the intestinal mucosa of ascending colon ulcer were taken for pathological analysis (Fig. 1B), which can help to make a clear diagnosis.

### 3.5 Endoscopic Mucosal Biopsy and Pathology

**Figure 4:**
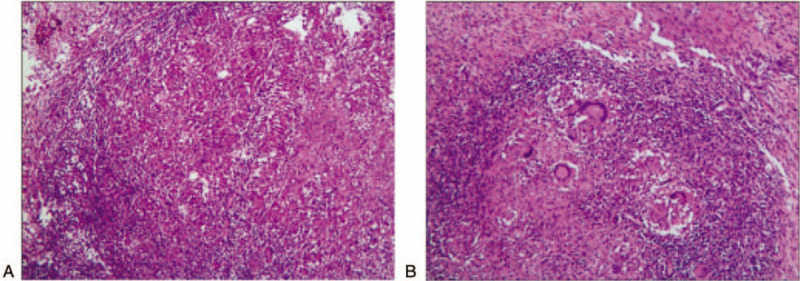
(A) Ileocecal full-thickness chronic granulomatous inflammation with coagulative necrosis, epithelial like cell proliferation and multi nuclear giant cell response. (B) The ileocecal part of the intestine is full-thickness chronic suppurative inflammation with abscess formation and local ulcer formation. Granulomatous inflammation of the whole intestinal wall accompanied by focal necrosis and a large number of multi nucleated giant cells hyperplasias.

All of the 10 patients underwent enteroscopy, and 8 cases underwent enteroscopic mucosal pathology at the same time, including 2 cases who underwent abdominal mass resection and intraoperative biopsy. The main purpose of this study was to observe the formation of caseous granuloma, including some local manifestations, such as granuloma formation and multinucleated giant cells, as well as the accumulation of epithelial-like cells (Fig. 4A). We analyzed a patient that underwent surgical resection of the ileocecal constricted mass. The mass is located in the proximal ileocecal part of ascending colon, about 10 × 6 cm in size. It is a new ulcerative vegetable like organism with hard texture. It infiltrates into the lateral abdominal wall of the form of plaques. The proximal intestine of the tumor is swollen and hypertrophic with chronic obstruction. The root of mesentery can reach the enlarged lymph nodes, with a larger diameter of about 1.5 cm. At the same time, the pathological analysis of the right colon was made (Fig. 4B). They are shown as Table 4.

**Table 4:**
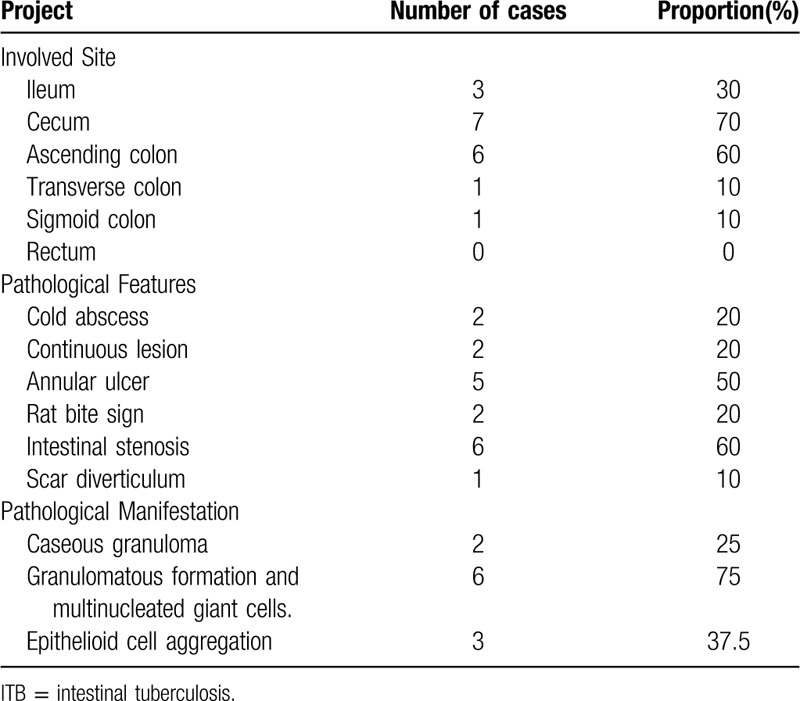
Endoscopic and pathological features of 10 patients with ITB.

### 3.6 Treatment Outcomes for Diagnosis

10 patients with ITB received medical treatment. Laparoscopic surgery was performed and progressive anti-tuberculosis therapy was performed after operation. 2 cases of abdominal pain with colon and stomach were the primary symptoms. After initial diagnosis in our hospital, all patients went to the tuberculosis hospital to receive standardized ITB treatment, 12 to 18 months, and 3 or 4 times intensive treatment were treated for 3 months, followed by consolidation therapy for 9 to 15 months. After 6 weeks of anti-tuberculosis treatment, the symptoms of abdominal pain, diarrhea, fever and night sweats were significantly improved. Regular blood examination, liver and kidney examination and colonoscopy were performed in patients 6 to 12 months after standard anti-tuberculosis treatment. The clinical symptoms were obviously relieved or completely disappeared. The colonic lesion was also significantly improved by colonoscopy, and the lesion area was significantly reduced or completely disappeared. There was no recurrence.

## 4 Discussion

According to our knowledge, there is a lack of effective etiological diagnosis in the ITB. The diagnosis of ITB is carried out in patients with appropriate clinical manifestations.^[8–10]^ Our study confirmed that the common clinical symptoms of ITB are abdominal pain, weight loss, nausea or vomiting, changes from stool habits, etc., which are consistent with many proven literatures.^[11]^ Clinically, patients with ITB are more likely to develop low fever, night sweats and potential respiratory symptoms than patients with IBD.^[12,13]^ Patients with Crohn are more likely to have hematochezia than those with ITB.^[14,15]^ In our study, 20% of the patients have clinical symptoms of bloody stool, which is different from the clinical manifestations of typical ITBs,^[16]^ which is noteworthy.

The 10 patients in our study received colonoscopy. We found and emphasized the importance of endoscopy and mucosal biopsy in the diagnosis of these patients. The positive rate of TB diagnosis specific method is relatively low.^[17]^ For patients that do not meet the diagnosis consensus standard,^[18–20]^ enteroscopy should be completed in the early stage. For the atypical signs of enteroscopy, on the basis of excluding CD, anti-tuberculosis therapy treatment should be tried as early as possible. If possible, colonoscopy should be performed in all patients with ITB because it can directly show mucosal lesions and stenosis, and allows histologic biopsy and tuberculosis culture.^[21,22]^ Most of the lesions collected were located in the ileocecum at the distal end of the small intestine, and the main lesions were intestinal stenosis and annular ulcers at the end of the ileum. These observations are consistent with previous reports on ITB using enteroscopy or enterography.^[22,23]^ The main manifestations of ITB under enteroscopy were ileocecal mucosal congestion, edema, ulcer formation, inflammatory polyps and intestinal stenosis.^[24]^ Among them, a small number of patients with cold abscess (20%) and scar diverticulum (10%) were found under enteroscopy, which was an atypical sign of the previously less described ITB.^[25–27]^ If endoscopy is not considered to be characteristic of ITB, then multiple biopsies from histopathological or bacteriological studies will not be performed, nor will specific treatment trials be conducted. Therefore, as shown in this study, the results of endoscopy are very important to the diagnosis of ITB in patients with few or no clinical symptoms.^[28]^ Biopsy can find granuloma, caseous necrosis, acid-fast bacilli and so on. Among the cases we collected, 2 patients underwent endoscopic examination and surgical treatment at the same time. It can be inferred that the early start of targeted medical intervention of ITB may help to prevent surgical complications. And tuberculous obstruction is considered to be the most common complication (24%) and has a high incidence, especially in developing countries.^[29,30]^ Therefore, it is very important to identify ITB patients and initiate early treatment. Early diagnosis and early treatment can effectively avoid the complications of ITB.

The CD index under endoscope has been well established.^[31,32]^ However, there is a lack of endoscopic instruments for the evaluation of ITB. Aoki et al^[33]^ were the first people to depict endoscopic features. Since this early series of reports, another case of colonoscopy diagnosis has emerged.^[34]^ Endoscopy usually shows an increase in the number of annular ulcers and granulomas in patients with ITB, while patients with Crohn's disease usually have more “jumping or segmental” lesions throughout the gastrointestinal tract. The typical longitudinal ulcers and pebble-like appearance changes.^[35–37]^ Histopathological findings, including caseous granuloma, caseous necrosis, or the discovery of acidfast bacilli in the intestinal mucosa, may be used in the diagnosis of ITB. The typical pathological changes of ITB often appear in the intestinal submucosa, such as caseous granuloma, caseous necrosis of intestinal wall layer or necrosis of intestinal lymph nodes, atrophy or stenosis of intestinal submucosa. Pathological examination has been recognized as the gold standard for the diagnosis of ITB.^[38]^ However, according to clinical experience, the typical pathological changes of ITB cases are rare. Most of them showed chronic inflammation or other nonspecific changes.^[39]^ Recently, a predictive model for the differential diagnosis of ITB and CD has been established.^[40]^ For clinicians, it is also important to distinguish clinical, radiologic and endoscopic features to distinguish some easily misdiagnosed diseases such as ITB and CD. Therefore, we suggest that clinicians should highly doubt ITB, in patients with abdominal symptoms from areas with high incidence of tuberculosis in order to prevent complications caused by delayed diagnosis. Diagnostic methods such as CT, colonoscopy and laparoscopy should be combined, and appropriate tissues should be obtained for histological confirmation and tuberculosis culture.

Although this study is retrospective and the sample size is small, we pay more attention to the role of endoscopy and pathological examination in the early diagnosis of ITB. The results of endoscopic examination showed that the lesions mainly involved ileocecal and ascending colon, with the characteristics of rat bite and annular ulcer. This is consistent with the results reported in the literature.^[41]^ From a pathological point of view, fissure ulcers, non-caseous granuloma and lymphocyte aggregation support the diagnosis of CD. Caseous necrosis of the intestinal wall and lymph nodes are more common to ITB. Caseous necrosis was only seen in ITB. Fissure ulcer and caseous necrosis are of great significance in differential diagnosis, but there are few typical above-mentioned manifestations of this group of patients. Although pathological examination has been recognized as the gold standard for the diagnosis of ITB. However, due to clinical experience, cases of ITB characterized by typical pathological changes are rare. Most of them showed chronic inflammation or other non-specific changes. The main reason is that both CD and ITB are chronic inflammation of the whole intestinal wall and the depth of the diseased tissue is not enough. The sensitivity of endoscopic biopsy specimens to typical lesions was significantly lower than that of surgical specimens. This may be due to the limitations of examination methods, and endoscopic biopsies usually obtain fewer tissue samples.

## 5 Conclusion

To sum up, ITB lacks specific clinical features, endoscopic observation and pathological biopsy is a more reliable method of diagnosis. Strengthening the analysis and summary of the characteristics of colonoscopy plays an important role in the diagnosis and treatment of this disease. Clinicians can carry out comprehensive diagnosis and treatment of tuberculosis on the basis of enteroscopy and pathology, clinical manifestations of tuberculosis, T cell examination of tuberculosis, X-ray, high resolution CT, of chest and abdomen and diagnostic anti-tuberculosis treatment.

## Acknowledgments

This work was supported by gastroenterology center of Changzhou Second People's Hospital.

## Author Contributions

The manuscript was written through the contributions of all authors. All authors have given approval to the final version of the manuscript.
